# Patterns of livestock depredation and cost‐effectiveness of fortified livestock enclosures in northern Tanzania

**DOI:** 10.1002/ece3.5644

**Published:** 2019-09-15

**Authors:** Bernard M. Kissui, Christian Kiffner, Hannes J. König, Robert A. Montgomery

**Affiliations:** ^1^ The School for Field Studies Center for Wildlife Management Studies Karatu Tanzania; ^2^ Institute of Land Use Systems Leibniz Centre for Agricultural Landscape Research (ZALF) Müncheberg Germany; ^3^ Research on the Ecology of Carnivores and their Prey Laboratory Department of Fisheries and Wildlife Michigan State University East Lansing MI USA

**Keywords:** African lion, conservation intervention, cost‐benefit analysis, human–carnivore conflict, livestock enclosure, spotted hyena

## Abstract

Human–carnivore conflicts and retaliatory killings contribute to carnivore populations' declines around the world. Strategies to mitigate conflicts have been developed, but their efficacy is rarely assessed in a randomized case–control design. Further, the economic costs prevent the adoption and wide use of conflict mitigation strategies by pastoralists in rural Africa. We examined carnivore (African lion [*Panthera leo*], leopard [*Panthera pardus*], spotted hyena [*Crocuta crocuta*], jackal [*Canis mesomelas*], and cheetah [*Acinonyx jubatus*]) raids on fortified (*n* = 45, total 631 monthly visits) and unfortified (traditional, *n* = 45, total 521 monthly visits) livestock enclosures (“*bomas*”) in northern Tanzania. The study aimed to (a) assess the extent of retaliatory killings of major carnivore species due to livestock depredation, (b) describe the spatiotemporal characteristics of carnivore raids on livestock enclosures, (c) analyze whether spatial covariates influenced livestock depredation risk in livestock enclosures, and (d) examine the cost‐effectiveness of livestock enclosure fortification. Results suggest that (a) majority of boma raids by carnivores were caused by spotted hyenas (nearly 90% of all raids), but retaliatory killings mainly targeted lions, (b) carnivore raid attempts were rare at individual households (0.081 raid attempts/month in fortified enclosures and 0.102 raid attempts/month in unfortified enclosures), and (c) spotted hyena raid attempts increased in the wet season compared with the dry season, and owners of fortified bomas reported less hyena raid attempts than owners of unfortified bomas. Landscape and habitat variables tested, did not strongly drive the spatial patterns of spotted hyena raids in livestock bomas. Carnivore raids varied randomly both spatially (village to village) and temporally (year to year). The cost‐benefit analysis suggest that investing in boma fortification yielded positive net present values after two to three years. Thus, enclosure fortification is a cost‐effective strategy to promote coexistence of carnivores and humans.

## Graphical abstract

Carnivore raid attempts at individual households are quite rare, but fortified bomas are effective in reducing carnivore raids. Spotted hyena was the main carnivore raiding livestock bomas, and the raids increased in the wet season compared with the dry season. Owners of fortified bomas reported less hyena raid attempts than owners of unfortified bomas. None of the landscape and habitat variables tested appeared to strongly drive the spatial patterns of spotted hyena raids in livestock bomas. Livestock boma fortification is cost‐effectiveness strategy to promote carnivores and human coexistence.

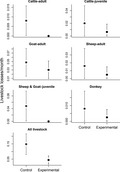

## 1 INTRODUCTION

Carnivore conservation efforts are facing substantial challenges across the globe, due to conflicts with people that arise from carnivore depredation of livestock (Dickman, 2010; Eshete, Marino, & Sillero‐Zubiri, 2017; Sutton et al., 2017). When carnivores kill livestock, people frequently retaliate against carnivores perceived to be responsible for the losses (Barlow, Greenwood, Ahmad, & Smith, 2010; Kissui, 2008), thus threatening the persistence of carnivore populations (Patterson, Kasiki, Selempo, & Kays, 2004; Woodroffe & Ginsberg, 1998). At the same time, livestock depredation strongly affects the quality of peoples' livelihoods (Constant, Bell, & Hill, 2015; Eshete et al., 2017; Mkonyi, Estes, Msuha, Lichtenfeld, & Durant, 2017). Therefore, mitigating these conflicts is an essential carnivore conservation goal, particularly in human‐dominated landscapes to promote human–carnivore coexistence. Coexistence between carnivores and humans is influenced by ecological, socioeconomic, and political variables (Eshete et al., 2017; Lute, Carter, Lόpez‐Bao, & Linnell, 2018; Mkonyi et al., 2017). Despite these complexities, evidence suggests that human and carnivores can coexist in landscapes where proper incentives, peoples' values, legislation, and appropriate conflict mitigation practices (technical, educational and stakeholder dialog) are promoted and implemented (Carter, Shrestha, Karki, Pradhan, & Liu, 2012; Chapron et al., 2014; Van Eeden et al., 2017; Schuette, Creel, & Christianson, 2013). Ideally, conservation management aimed at sustainable human–carnivore coexistence should be based on landscape‐level planning (Di Minin et al., 2016) whereas priority areas for carnivore conservation actions are identified based on a practical and science‐based framework (Van Eeden et al., 2017).

Riggio et al. (2013) estimated that Tanzania contained more than 40% of Africa's remaining lion (*Panthera leo*) population. The Maasai steppe in northern Tanzania is an important area for large carnivores and a potential lion stronghold (Riggio et al., 2013). The region is also home to considerable populations of spotted hyenas (*Crocuta crocuta*), leopards (*Panthera pardus*), cheetahs (*Acinonyx jubatus*), African wild dogs (*Lycaon pictus*), and black‐backed jackals (*Canis mesomelas*). However, these carnivores and their natural prey face substantial pressures from the increasing human population and ongoing land use changes including conversion of rangelands into agricultural farms, as well as increased settlements, and infrastructure (Bond, Bradley, Kiffner, Morrison, & Lee, 2017; Hariohay, 2013; Kiffner et al., 2017; Msoffe et al., 2011). The Maasai steppe is also characterized by seasonal migration of several wildlife species (e.g., wildebeest [*Connochaetes taurinus*] and zebra [*Equus quagga*]) which move seasonally between human‐populated areas, multiple‐use areas, and fully protected areas (Kiffner, Nagar, Kollmar, & Kioko, 2016). Large carnivores may follow the movement of some of their main prey species (e.g., leaving fully protected areas during the wet season and thus coming in frequent contact with livestock and people), and these seasonal movements may contribute to high levels of human–carnivore conflicts and retaliatory killing of carnivores (Koziarski, Kissui, & Kiffner, 2016).

Livestock predation contributes to indiscriminate retaliatory killing of carnivores and their precipitous global decline (Ripple et al., 2014). Central to reducing retaliatory carnivore killings is the implementation of human–carnivore conflict mitigation strategies. However, few studies have provided quantitative assessments of the efficacy of various strategies used in carnivore conflict mitigation (Eklund, López‐Bao, Tourani, Chapron, & Frank, 2017). In a recently published review, van Eeden et al. (2018) also reported a general scarcity of rigorous experimental designs and lack of quantitative comparisons for conflict mitigation interventions due to poor experimental controls. Both issues hamper proper inference about effectiveness of human–carnivore conflict mitigation strategies. Fortification of livestock enclosures (known locally as “*bomas*”) with sturdy fences using posts and chain‐link fencing is one of the predominant intervention methods used to prevent livestock depredation at night (Lichtenfeld, Trout, & Kisimir, 2015; Manoa & Mwaura, 2016; Okello, Bonham, & Hill, 2014; Sutton et al., 2017; Weise et al., 2018). In this study, we use a randomized case–control experimental design of longitudinal data collected over a five‐year period to examine the effectiveness of boma fortification in reducing carnivore raids on livestock. And, we include a novel dimension; an assessment of how landscape and habitat variables might also influence effectiveness of boma fortification in reducing livestock depredation by carnivores. We structure the analysis via the pursuit of five models (Table 1), each representing an a priori hypothesis about the effects of landscape and habitat variables on livestock depredation risk in bomas by large carnivores. Landscape and habitat variables are known to influence site selection where carnivores hunt and kill prey at different scales (Davidson et al., 2012), and hunting success by carnivores is associated with fine‐scale spatial features such as proximity to rivers (Hopcraft, Sinclair, & Packer, 2005). Linear and man‐made landscape features such as roads have also been shown to affect the distribution and activity pattern of carnivores because carnivores use roads to travel across landscapes (Raitera, Hobbsb, Possinghamd, Valentineb, & Proberb, 2018). Consequently, we predicted that bomas close to rivers, roads, and protected areas would be likely to experience more predation than those faraway from these features. We also predicted that bomas located in areas with high NDVI and little agriculture should experience increased livestock predation risk because high NDVI is associated with high vegetation productivity which may increase local prey densities and support predators (Petorelli, Bro‐Jørgensen, Durant, Blackburn, & Carbone, 2009). Little agriculture may indicate fewer disturbances by people which could support habitat use by carnivores. For the effect of season, we predicted that more livestock predation in bomas would occur during the wet season due to extensive movement of wildlife from protected area into communal land as reported from previous studies by Kissui (2008) and Koziarski et al. (2016). Quantifying the long‐term spatiotemporal dynamics of carnivore‐human conflicts would provide vital information to effectively inform conflict mitigation strategies across a landscape (Teichman, Cristescu, & Nielsen, 2013). Monitoring livestock depredation by carnivores over longer time periods and in a spatially explicit way make it feasible to develop risk maps to identify and predict hotspots of human–carnivore conflict (Weise et al., 2018). This could be particularly useful to direct implementation of mitigation strategies in a cost‐effective manner.

**Table 1:** A priori generalized linear mixed models representing hypotheses concerning the effects of spatiotemporal covariates on: (a) hyena raid attempts and (b) hyena successful raids on livestock bomas

Variables	*df*	AICc	ΔAICc	*wi*
*(a) Attempts*				
Boma type + Season	5	645.71	0	0.86247
Distance to river + Distance to road + Distance to PA + Proportion agriculture + NDVI +Season	9	650.12	4.41	0.09509
Distance to river + Distance to road + Distance to PA + Proportion agriculture + NDVI +Season + Boma type	10	651.74	6.03	0.04230
Distance to river + Distance to road + Distance to PA + Proportion agriculture + NDVI	8	663.75	18.04	0.00010
Boma type	4	665.85	20.14	0.00004
*(b) Success*				
Boma type	4	352.99	0.00	0.56046
Boma type + Season	5	354.56	1.57	0.25504
Distance to river + Distance to road + Distance to PA + Proportion agriculture + NDVI	8	356.08	3.09	0.11934
Distance to river + Distance to road + Distance to PA + Proportion agriculture + NDVI +Season	9	358.08	5.09	0.04402
Distance to river + Distance to road + Distance to PA + Proportion agriculture + NDVI + Season + Boma type	10	359.54	6.56	0.02114

Though several carnivore species depredate livestock in the Maasai steppe, spotted hyenas cause the majority of depredation on small‐sized livestock (goats and sheep; Koziarski et al., 2016; Mkonyi et al., 2017). As high livestock depredation risk correlates with high probabilities of human retaliatory killing of carnivores (Kissui, 2008), we thus aimed to assess the impact of retaliatory killings on different carnivore species involved in livestock depredation. We hypothesized that carnivore species causing high levels of livestock depredation should suffer higher impact of retaliatory killings.

Based on our long‐term experience in the Maasai steppe, working with pastoralist communities to implement boma fortification as a strategy to reducing livestock depredation by carnivores, and based on studies conducted elsewhere in Africa such as by Weise et al. (2018), we found the economic costs associated with the construction of predator‐proof bomas to be one of the important challenges against adoption and wide use of conflict mitigation strategies by livestock keepers in rural Africa. Using the long‐term dataset on the costs associated with the construction of predator‐proof bomas, we assessed whether boma fortification using chain‐link fences was a worthwhile investment by the individual livestock keepers using a cost‐benefit analysis to quantify the net present value of the conservation investment. With this analysis, it will be possible to properly advise livestock keepers about the long‐term economic benefits of boma fortification and thus enhance acceptance and wide use of this strategy for reducing human–carnivore conflicts and promoting coexistence between human and carnivores in human‐dominated landscapes.

Therefore, the specific objectives of this study were to: (a) assess the extent of retaliatory killings of major carnivore species due to livestock predation, (b) describe the spatiotemporal characteristics of carnivore raids on livestock enclosures, (c) analyze whether spatial covariates influenced livestock depredation risk in livestock enclosures, and (d) examine the cost‐effectiveness of fortified enclosures in preventing livestock depredation.

## 2 MATERIALS AND METHODS

### 2.1 Study area

**Figure 1:**
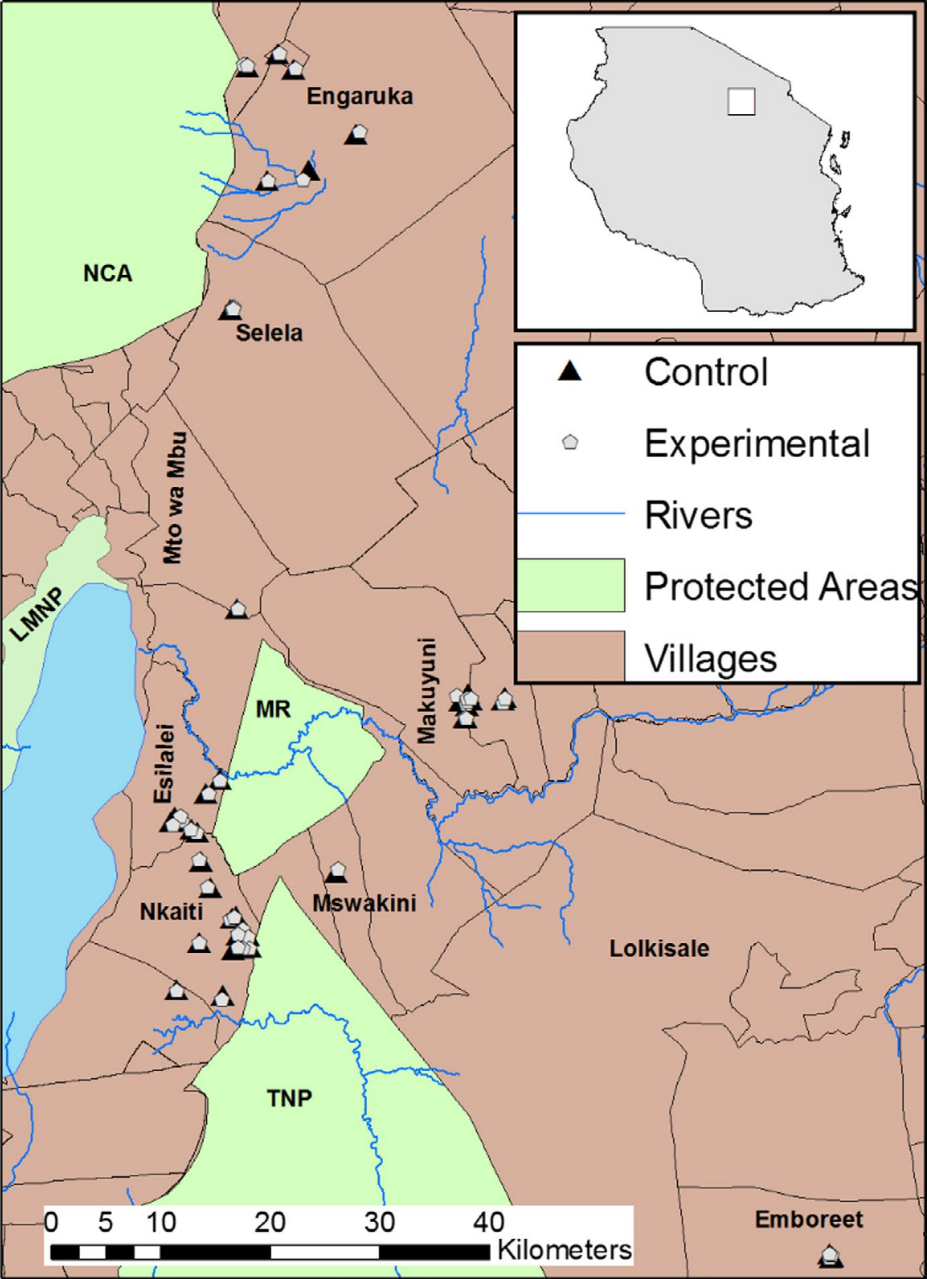
Map of the study area showing the location of protected areas, experimental and control bomas

The Maasai steppe of northern Tanzania covers some 30,000 km^2^ (Figure 1) with the altitude ranging from 1,000 m to 2,600 m above sea level and annual rainfall between 500 mm and 650 mm. The short rains fall from October to December and long rains falling from March to May (Prins & Loth, 1988). The ecosystem is renowned wet season migration of abundant ungulates including wildebeest (*Connochaetes taurinus*), zebra (*Equus quagga*), buffalo (*Syncerus caffer*), and elephant (*Loxodonta africana*) from protected areas to dispersal lands interspersed among the human communities (Bond et al., 2017; Borner, 1985; Kiffner et al., 2016; Lamprey, 1964; Morrison & Bolger, 2014). The core protected areas in the steppe are Tarangire and Lake Manyara National Parks (NP). While most wildlife species in Lake Manyara NP are considered to be residents, Tarangire NP serves as a key dry season range for many migratory species (Morrison & Bolger, 2014).

Beside wildlife conservation, livestock keeping and agriculture are major land uses in the Maasai steppe. Livestock, including cattle, goats and sheep, and donkey form the majority of the grazing biomass in the Simanjiro plains (Mwalyosi, 1992) and livestock densities usually exceed those of wildlife species in areas outside fully protected areas (Kiffner et al., 2016). The Maasai steppe has been experiencing increasing pressure associated with expanding agriculture from small scale subsistence farming to large scale commercial farming (Borner, 1985; Oikos, 2002). Other forms of land use changes and economic development activities include human settlements driven by immigration and tourism‐related opportunities (Msoffe et al., 2011).

### 2.2 Field methods

We collected field data from 2009 to 2013 as part of the long‐term human–carnivore conflict monitoring and mitigation strategies by the Tarangire Lion Project. Data on carnivore depredation attempts and successful livestock attacks in bomas were collected using a paired experimental–control design. Bomas fortified with chain‐link fences (referred to as experimental bomas) were paired with a nearby, unfortified traditional boma constructed using thorn bushes (referred to as control boma). Experimental bomas were randomly included in the study based on the construction date which was used as a starting date for data collection and monitoring of depredation events. The geographical coordinates of both experimental and control bomas were recorded using a Garmin GPS device. Bomas from thirteen villages (Emboreet, Engaruka, Esilalei, Makuyuni, Minjingu, Mswakini, Naiti, Olasiti, Oltukai, and Selela) were included in the study across the study area (Figure 1). For each constructed boma, we recorded the size of the boma (circumference), number of chain‐link fences used, type and number of poles, and cost of construction.

Each experimental–control pair of bomas was monitored through revisits on a thirty‐day interval, and a short semistructured interview was conducted with the owners and/or adult residents of each household. Where it was not possible to conduct regular monthly visits, carnivore raids information were collected from boma owners targeting the last thirty days of the ending or just ended month. The interviews were designed to collect information on carnivore species raiding the bomas and whether the carnivores were successful in breaking or jumping over the boma wall. The species of carnivores involved in boma raids were identified by respondents based a combination of actual sightings of predators, using tracks and signs based on the commonly known characteristic patterns and behavior of raiding predator which differ among the major carnivore species found in the landscape. Although interviews are commonly used techniques in studies of human–carnivore conflicts (e.g., Kolowski & Holekamp, 2006; Koziarski et al., 2016; Mkonyi et al., 2017; VanBommel, Bij De Vaate, De Boer, & De Longh, 2007; Woodroffe, Frank, Lindsey, Ole Ranah, & Roman, 2007), this style of data collection can suffer from bias related to respondent's memory. For this study, it is possible that respondents could have misidentified the carnivore species involved in boma raids and thus may have introduced bias. However, our survey strategy of collecting boma raid information specifically for the most recent events (i.e., last thirty days) might have reduced bias and improved the quality and reliability of the data. For each visit, we recorded information on carnivore species involved in boma raids, type and number of livestock attacked by the raiding predator and the fate of the predator (whether or not the attacking predator was killed or injured by people in retaliation to livestock attack). Data on retaliatory killings of carnivores were collected using diary records by field personnel permanently residing in respective villages included in the study (Kissui, 2008).

For spatial analyses, we used the boma (i.e., household) as the unit scale of analysis (Montgomery, Hoffmann, Tans, & Kissui, 2018) and developed a geographic database in ArcMAP 10.3.1 (ESRI) where all covariates were represented as rasters across the extent of the study area. With data downloaded from the Food and Agriculture Organization (FAO) of the United Nations, we were able to calculate distance to rivers and roads at 30 m resolution. All distance rasters were calculated as cost distances where barriers to carnivore movement were represented by the large lakes (e.g., Lake Manyara, Lake Natron, and Lake Eyasi) of the region. Thus, the resultant distance rasters represented the distance that an animal would need to travel to move around these obstacles. We also mapped the proportion of agricultural land (again depicted by FAO data) within a network of 250 m grid cells throughout the study area. The distances of each boma to the nearest protected area were calculated at a 30 m resolution. We tracked longitudinal changes in vegetation using the normalized difference vegetation index (NDVI) data managed by USGS as proxy for vegetation cover. We downloaded NDVI raster data, represented at a 250 m resolution, in the nearest 3‐month period to the data in which we tracked carnivore attempts to livestock depredation and successful attacks. For temporal data, we defined the wet season to be from November to May and dry season from June to October of each year.

### 2.3 Data analysis

#### 2.3.1 Effects of spatiotemporal covariates on livestock boma raids

Because the data for this study were collected via repeated visits to each experimental and control boma, only bomas with at least three revisits were included in the analysis. Due to small sample size of depredation attempts and successful attacks for most individual carnivore species, we restricted the spatiotemporal analyses to spotted hyena depredation only. To test the effect of spatial patterns, we conducted two separate analyses, for *attempted* and *successful* hyena raids in bomas. We constructed five a priori candidate models representing hypotheses concerning the effects of spatiotemporal covariates on hyena raid attempts and successful raids on livestock bomas (Table 1). The candidate models were created based on the available data, knowledge of the study population, and on the biological plausibility of an a priori hypothesis (Burnham & Anderson, 2002). We used an information‐theoretic approach (Burnham & Anderson, 2002), with Akaike's Information Criterion (AIC) to select the top‐ranking model.

We analyzed the effects of the spatiotemporal variables on boma raids using generalized linear mixed models with binomial error distribution using R 3.3.2 (R Core Team, 2014). The response variables were defined as to whether a particular boma experienced any hyena raid attempt (coded as 1) or no raid attempt (coded as 0) within a thirty‐day period. Similarly, successful attacks on livestock in a particular boma were coded as 1 if hyenas succeeded in attacking (injured or killed) livestock and coded as 0 if no livestock was attacked within a thirty‐day period. To account for spatial and temporal nonindependence across bomas, we allowed the models to have random intercepts for the different villages and survey years.

**Figure A1:**
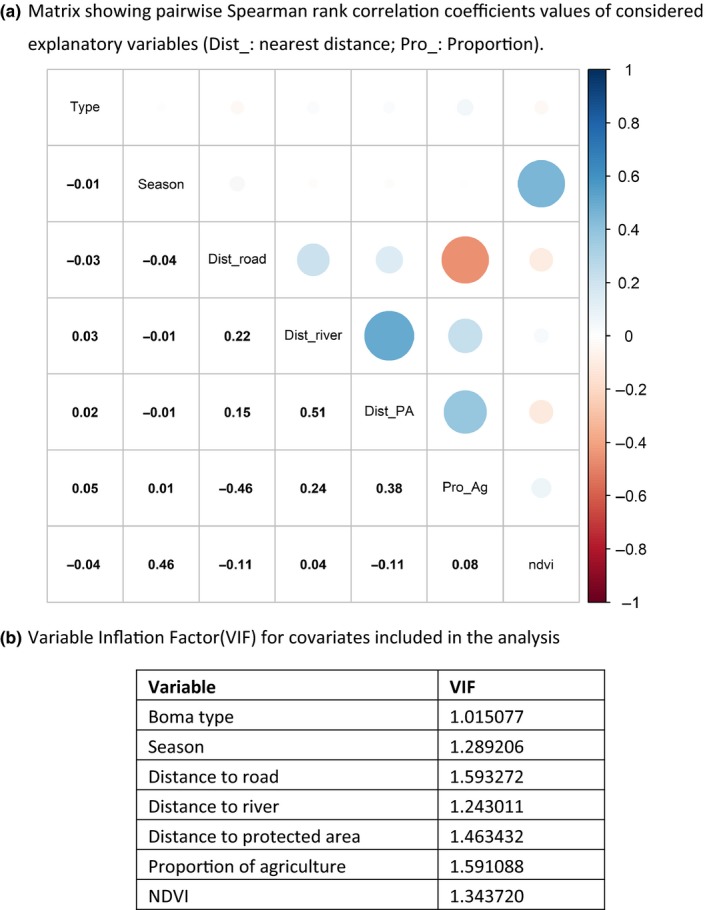
Matrix showing pairwise Spearman rank correlation coefficients values of considered explanatory variables (Dist_: nearest distance; Pro_: Proportion)

**Figure A2:**
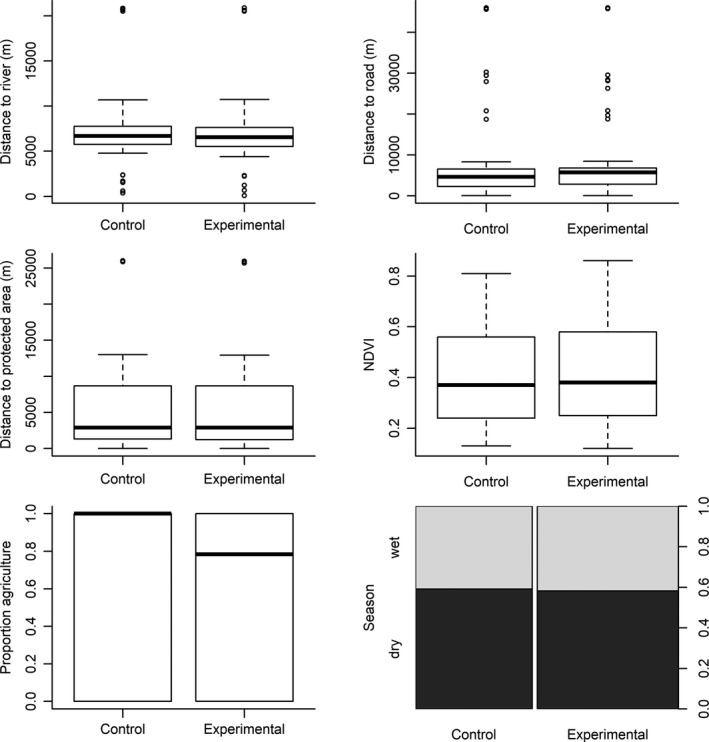
Range of values for landscape variables for experimental and control bomas

Before model fitting, we tested for collinearity among all explanatory variables using the *corrplot* package (Wei & Simko, 2017) and calculated the variable inflation factor (VIF) using the *usdm* package (Naimi, 2017). We found no strong correlations among variables, and the VIF were all found to be less than 2.5 suggesting that multicollinearity should not be a concern for the analysis (Figure A1 and Table A1). Within explanatory variables, the range of values was similar between control and experimental bomas (Figure A2), lending further evidence for a well‐balanced experimental design. To compare the likelihood of attack attempts and successful attacks for fortified and unfortified bomas (and other explanatory variables), we converted the logistic regression coefficients into odds ratios by taking the exponent of the regression estimates (Crawley, 2005).

#### 2.3.2 Cost‐benefit analysis of fortified bomas

To assess whether fortifying bomas is a cost‐effective investment (e.g., Sutton et al., 2017), we conducted a cost‐benefit analysis (CBA: Bergen, Löwenstein, & Olschewski, 2002). In an effort to quantify the net present value of the conservation investment, we first calculated the cash flow as the mean differences in annual depredation rates for each livestock species (and age class) between control and experimental bomas. Annual depredation rates were multiplied with the average local price (in Tanzanian Shillings) for each livestock head (adult/juvenile cattle: US $ 364/157; adult donkey: US $ 80; and adult/juvenile sheep or goat: US $ 42), obtained from livestock markets in the study area and converted to US dollars. In accordance with common CBA practices (Bergen et al., 2002), we discounted annual cash flows with the true interest rate, that is the nominal interest rate minus inflation rate. For the nominal interest rate (9.18%), we used the interest rate of a 5‐year fixed‐rate treasury bond (Bank of Tanzania, 2018) and used the average inflation rate from 1999 to 2018 (7.2%) to control for price increases (Trading Economics, 2018). To account for uncertainty in the used interest rate (1.98%), we also conducted sensitivity analyses and considered interest rates of 5, 10, and 15%. Costs for boma fortification varied from US $ 77 to 1509 (average of US $ 186) largely due to differences in boma size and the type of materials used in boma construction (wooden vs. metal poles). Thus, we estimated net present values for small (circumference of 15–30 m), average (circumference 45–60 m), and large‐sized bomas (circumference > 75 m) and for four different interest rate scenarios (1.98%, 5%, 10%, and 15%) and considered five years as the project duration. Thus, our CBA approach includes a sensitivity analysis for various relevant scenarios.

## 3 RESULTS

### 3.1 Characteristics of carnivore raids in bomas

**Figure 2:**
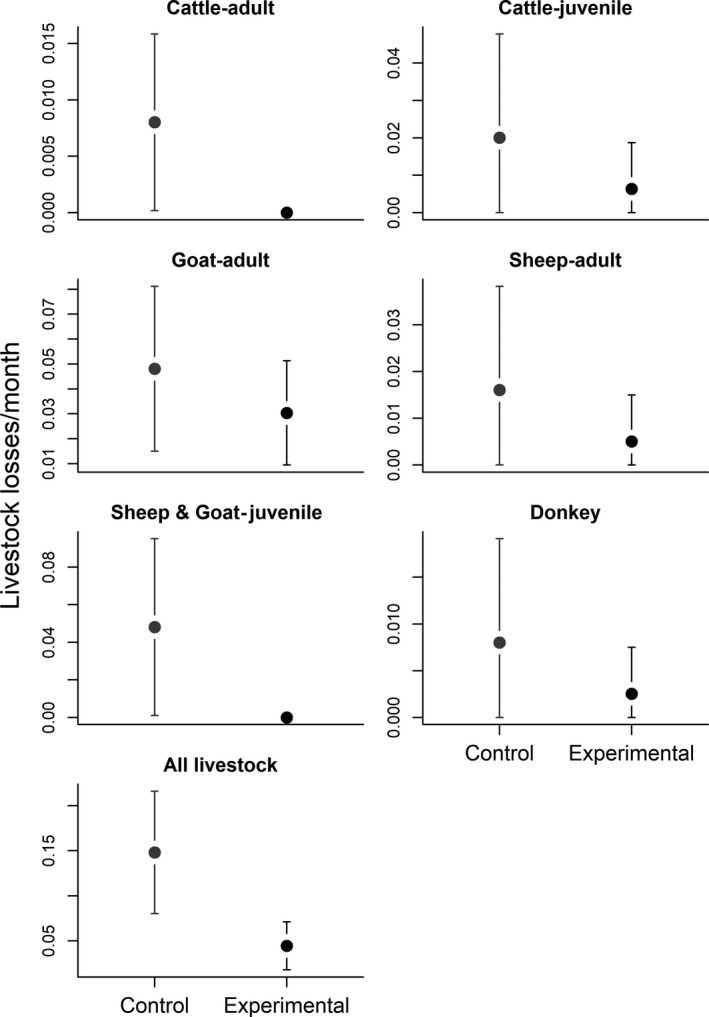
Averages and associated 95% confidence intervals of monthly livestock losses separated by livestock species and age class, (and for all livestock type combined) due to large carnivores (African lions, spotted hyenas, leopards, cheetahs, and black‐backed jackals) predation in control (traditional bomas) and experimental (fortified) bomas in northern Tanzania

A total of 90 bomas of which 50% (*n* = 45) were fortified (i.e., experimental) and 50% (*n* = 45) were unfortified (i.e., control) were included in the study. We conducted 1,152 monthly revisits from 2009 to 2013; 631 revisits were conducted to experimental bomas and 521 revisits to control bomas. The average distance between experimental and control boma was 233.04 ± 28 m (Mean ± *SE*) with the distances ranging from 15 to 972 m. The average rates of monthly predation events by all carnivore species were higher in unfortified enclosures (0.102 raid attempts/month) than in fortified enclosures (0.081 raid attempts/month) whereas goats were the most frequently killed livestock species during these events (Figure 2).

#### 3.1.1 Retaliatory killing of carnivores

**Figure 3:**
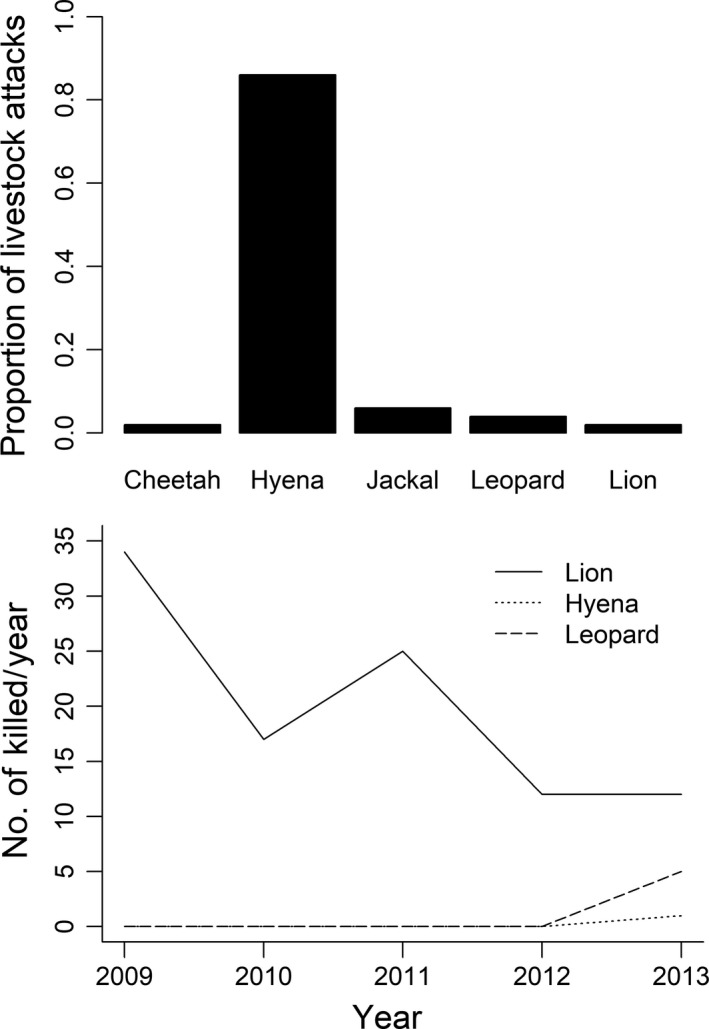
Proportion of livestock attacks caused by different carnivore species (Top graph) and number of individuals from different carnivore species killed each year by pastoralists in retaliation to livestock predation by these carnivores (Lower graph) in northern Tanzania

During the five‐year period (2009–2013) of this study (*n* = 172) livestock attack events (both attempts and successes) were recorded, and 89% were due to spotted hyena, 4.1% by leopard, 3.5% jackal, and 2.3% lions. Over the same period, we recorded (106 individual carnivores (lions, hyenas, and leopards) to have been killed by pastoralists in retaliation for livestock depredation. On average, 20 ± 4.2 (mean ± *SE*) lions were reportedly killed each year compared with 0.2 ± 0.2 (mean ± *SE*) spotted hyenas and 1 ± 1 (mean ± *SE*) leopards (Figure 3). Despite spotted hyenas being responsible for the majority of livestock depredation, lions were more likely to be killed in retaliation compared with spotted hyenas and leopards (Figure 3). No events of retaliatory killing of other carnivore species were recorded during this period.

#### 3.1.2 Spatiotemporal correlates for spotted hyena raid attempts

The model containing season and boma type was the best in predicting spotted hyena raid attempts in livestock bomas. Parameter estimates for the top‐ranking model indicated that the effect of season to be strong on spotted hyena raid attempts on livestock bomas, and that raid attempts increased in the wet season compared with the dry season; odds ratios indicated that boma raid attempts were three times more likely to occur in the wet season than in the dry season. Owners of fortified bomas reported less hyena raid attempts (0.87 times) than owners of unfortified bomas (Table 2a), even though the effect was weak.

**Table 2:** Parameter estimates and the 95% confidence intervals (CI) for the top‐ranking model for the effect of spatiotemporal variables on (a) spotted hyena raid attempts and (b) raid successes. The relative effect size of the parameters is indicated by the odds ratio

	Estimate	Lower CI	Upper CI	Std. error	*z*‐Value	*p*‐Value	Odds ratio
*(a) Attempts*							
Intercept	−2.9777	−3.7474	−2.2981	0.3249	−9.1650	≤.001	
Boma type (Experimental vs. control)	−0.1401	−0.5679	0.28926	0.2162	−0.6480	.5170	0.869
Season (wet vs. dry)	1.1009	0.6330	1.5941	0.2423	4.5430	≤.001	3.007
*(b) Successes*							
Intercept	−3.2342	−4.0298	−2.5047	0.3305	−9.786	≤.001	
Boma type (Experimental vs. control)	−0.2357	−0.8767	0.4043	0.3193	−0.738	.46	0.790

#### 3.1.3 Spatiotemporal correlates for spotted hyena raid successes on livestock bomas

The logistic regression model containing boma type was the best in explaining spotted hyena raid successes. Parameter estimates for the top‐ranking model indicated that fortified bomas reduced raid successes by 0.79 times in comparison to unfortified bomas (Table 2b). In both raid attempt and raid success models, the random intercepts suggested substantial year‐to‐year variation as well as some variation among villages in spotted hyena depredation on livestock bomas (Table A2).

#### 3.1.4 Cost‐benefit analysis of boma fortification

**Figure 4:**
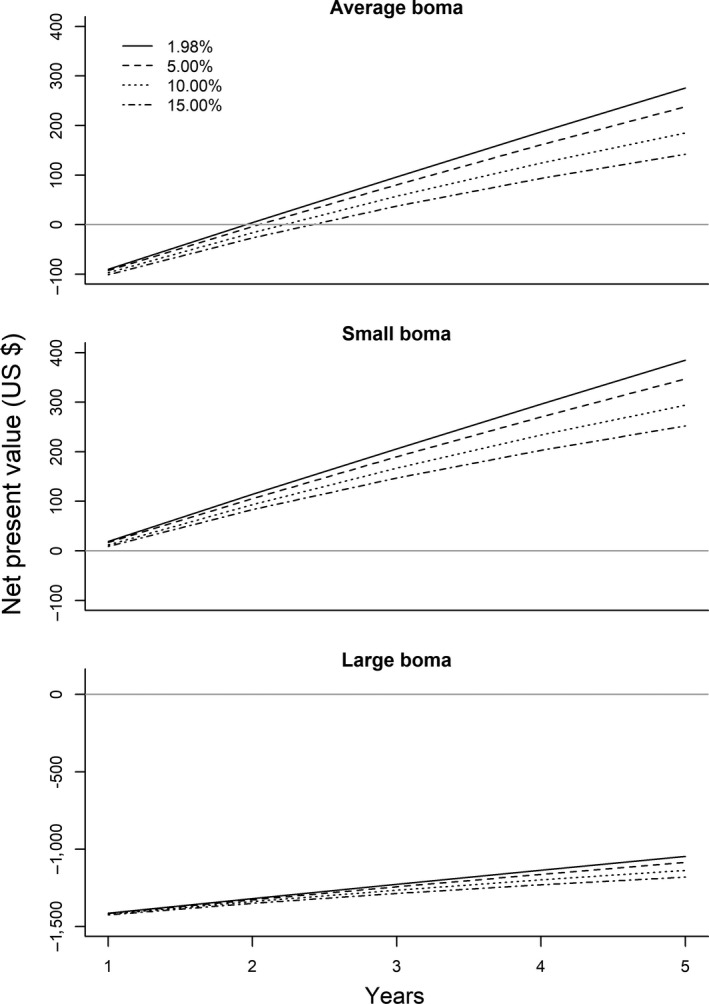
Net present value (US $) of boma fortification to reduce livestock depredation for average‐sized, small‐sized, and large‐sized bomas over a 5‐year time span and considering different interest rates. The intersection of the projected net present values and the gray line represents the break‐even point

For an average‐sized boma, cost‐benefit analyses suggest that investing in fortification results in a positive net present value after year two (1.98% interest rate) or latest in year three (interest rates > 5%; Figure 4). For small bomas, the net present value becomes positive in year one, irrespective of the considered interest rate. For large bomas (which require substantially more material and thus incur higher investment cost), the net present value remains negative even after five years. Only after 19 years, the net present value becomes positive (considering a 1.98% interest rate).

## 4 DISCUSSION

Via this longitudinal, quasi experimental study, we found that carnivore raids on specific livestock enclosures (bomas) do not occur daily, are primarily caused by spotted hyenas, and mainly occur during the wet season. Although some villages may be slightly more or less prone to spotted hyena raids, raids appear to occur rather randomly across the landscape and high‐risk areas cannot reliably be predicted by landscape variables. However, investing in fortified bomas appears to be a cost‐effective investment that pays off economically under most conditions.

### 4.1 Characteristics of carnivore raids in bomas and retaliatory killings of carnivores

Even though five carnivore species (African lion, leopard, spotted hyena, jackal, and cheetah) were recorded in raiding livestock bomas, spotted hyena was the species that caused the majority of the raid attempts and successful attacks on livestock. Thus, spotted hyenas appear to be much more capable of causing frequent depredation on livestock especially targeting goats and sheep, as recorded in previous studies in northern Tanzania (Kissui, 2008; Koziarski et al., 2016; Mkonyi et al., 2017; Mponzi, Lepczyk, & Kissui, 2014), in the Ethiopian highlands (Yirga et al., 2012), in Loitoktok, Kenya (Manoa & Mwaura, 2016) and in the Amboseli region, Kenya (Okello, Bonham, et al., 2014). The reasons for the high frequency of raids by spotted hyenas could be due to their ability to adapt to human‐dominated areas. Use of these landscapes might be facilitated by their predominantly nocturnal activity or commuting behavior (Hofer & East, 1993) which could allow them to travel long distances in a single night in search of foraging opportunities. Despite spotted hyenas being responsible for the majority of livestock depredation, lions appear to be disproportionally killed by local people in retaliatory killings (Figure 3). Various hypotheses including predator behavior, prey preferences, predator hunting strategies, and the cultural traditions of the local people have been proposed to explain the reasons for lions' being more vulnerable to retaliatory killing (Kissui, 2008). Whatever the underlying mechanisms are, high rates of retaliatory killings of lions are a substantial challenge for lion conservation in this landscape and need urgent conservation action involving pastoralist communities to promote changes in attitudes and increase tolerance (Dickman, 2010; Hazzah, Bath, Dolrenry, Dickman, & Frank, 2017; Mutanga, Vengesayi, Gandiwa, & Muboko, 2015; Odebiyi, Ayeni, Umunna, & Johnson, 2015). Ultimately, retaliatory killing of lions might increase livestock depredation, because spotted hyena populations may substantially increase when lion populations are reduced by human interventions (e.g., Green, Johnson‐Ulrich, Couraud, & Holekamp, 2018). Interestingly, our study found that carnivore raid attempts in livestock bomas did not occur daily at individual households, despite livestock depredation being perceived as a frequent problem among local residents (Koziarski et al., 2016). This could be driven by the discrepancy in the perceived versus actual carnivore depredation levels on livestock which are commonly reported in many studies (Kaartinen, Luoto, & Kojola, 2009; Woodroffe & Frank, 2005). Hence, “retaliatory” killing of lions may not only be driven by livestock depredation and economic motives, but may additionally be driven by cultural practices and even political motivations (Goldman, Pinho, & Perry, 2013; Ikanda & Packer, 2008).

### 4.2 Temporal patterns of livestock depredation

Spotted hyena raids on livestock at the bomas are highly influenced by season in the Maasai steppe, with most events occurring in the wet season. We acknowledge that our study system is highly migratory (Lamprey, 1964) with strong seasonal migration of dominant prey species including wildebeest, zebra, and buffalo from protected areas to dispersal areas in communal land. Thus, it is possible that the seasonal variation in livestock boma raids by spotted hyenas is influenced by these seasonal migrations of wild prey species, which may bring spotted hyenas into the community areas during the wet season, thus increasing encounter rate with livestock and people. We have found that lions from Tarangire NP also exhibit seasonal shifts in their home ranges—with wet season ranges being bigger and extending into communal areas (Kissui, 2013). Such seasonal variation in livestock depredation for both spotted hyenas as well as for lions was also recorded in a study by Kissui (2008) and by subsequent studies by Mponzi et al. (2014), Koziarski et al. (2016) and Mkonyi et al. (2017).

### 4.3 Spatial patterns of livestock depredation

It was expected that the landscape and habitat variables (rivers, roads, vegetation cover, and NDVI) to exhibit substantial effects on the likelihood of carnivore raids in bomas. However, our results found none of these features influenced the spatial patterns of spotted hyena raids in bomas in the Maasai steppe. Similar results were found by a study on human–black bear conflict in Canada (Porten, Cooper, Bickerton, & Salomon, 2014). We suggest that spotted hyena raids in bomas in Maasai steppe may be so widely spread across the landscape such that no single landscape variable or a set of variables can drive the spatial variability in raids pattern. Alternately, our findings may suggest that spotted hyena raids in bomas may be driven by other, unmeasured spatial variables or other drivers of spotted hyena behavior not considered in this study. Several studies on carnivore depredation on livestock (e.g., Karlsson & Johansson, 2010; Linnell, Odden, Smith, Aanes, & Swenson, 1999) have suggested the existence of “problem” individuals in carnivore populations that could lead to specific patterns in livestock depredation in a particular landscape. We suggest that the notion of problem carnivores in agropastoral landscapes in Africa requires additional investigation. We argue, for instance, that it is also possible that other fine‐scale landscape and habitat variables may exist that would explain the spatial pattern of spotted hyena raids in bomas better than the variable tested in the current study (Porten et al., 2014), which thus invokes the possibility of considering further research on carnivore raids in bomas at a finer scale than the one we have completed (Marucco & McIntire, 2010; Montgomery et al., 2018; Treves, Martin, Wydeven, & Wieden‐hoeft, 2011).

### 4.4 Cost‐effectiveness of boma fortification

Fortified bomas were found to reduce the likelihood of a successful raid by spotted hyenas and other carnivores. This result adds to increasing evidence in the body of literature that boma fortification in East African pastoralist communities can be an important conservation strategy for reducing livestock losses due to predation and used as a tool to promote human–carnivore coexistence. This is consistent with other studies by Schuette et al. (2013); Lichtenfeld et al. (2015); Okello, Kiringe, and Warinwa (2014); Manoa and Mwaura (2016); Sutton et al. (2017); Weise et al. (2018). Reduced livestock losses means that carnivores cause less obvious costs to local people and this may contribute to increased tolerance toward large carnivores.

Yet, our cost‐benefit analyses indicate that boma fortification, even under conditions of moderate livestock depredation rates (e.g., much higher depredation rates reported in areas adjacent to the Maasai Mara in Kenya by Sutton et al., 2017) can be a very profitable investment and requires rather modest monetary investment and should thus be a key element for strategies to reduce livestock losses due to carnivore depredation.

Even though fortified bomas reduce livestock depredation compared with unfortified bomas, fortified bomas are not zeroing livestock losses at night (see also Sutton et al., 2017). Most likely, this is because the effectiveness of fortified bomas is highly dependent on the condition of the fence and its regular maintenance for it to be effective (Weise et al., 2018). Additionally, this finding implies that no single human–carnivore conflict mitigation strategy is 100% effective in preventing carnivore attacks on livestock and stresses the need for pastoralists and conservationists to encourage application of multiple strategies and be willing to constantly improve the conditions of the interventions being applied for a successful outcome in reducing livestock depredation by carnivores.

## 5 CONCLUSION AND CONSERVATION IMPLICATIONS

Our analysis provides further quantitative evidence that livestock enclosure fortification is a cost‐effective conservation intervention that reduces livestock losses and thus effectively protects pastoral livelihoods and could promote human–carnivore coexistence in rural African landscapes.

In addition, this analysis shows that even in a landscape where human–carnivore conflicts are rather prevalent, it may be difficult to predict spatial patterns of conflicts based on landscape and habitat variables alone. Thus, dealing with human–wildlife conflicts implies integrating interdisciplinary knowledge of wildlife ecological data, socioeconomic information about land use, and relevant stakeholders. We suggest that looking at natural prey of carnivores and its movements could provide crucial information that can be used to describe spatiotemporal livestock depredation patterns. Thus, we recommend broadening the spectrum of target species in human–carnivore conflict studies to include prey species which will allow capturing a more holistic picture of interactions and depredation events in a landscape.

## CONFLICT OF INTEREST

None declared.

## AUTHOR CONTRIBUTION

B. K. developed the original research design, contributed in field data collection and preparation for data analysis and drafting the article; C.K., R.M., & H.K. contributed in carrying out statistical and spatial analyses of the data, editing and reporting results. All authors contributed substantially to the writing and proofing the article, and all have read and approved the final version of the submitted article.

## Data Availability

The boma data used in this study have been deposited to a publicly accessible Dryad Digital Repository (https://datadryad.org//; Kissui, Kiffner, König, & Montgomery, 2019). The spatial data are already publicly available through website: https://earthexplorer.usgs.gov).

## APPENDIX 1

**Table A1:** Variable inflation factor (VIF) for covariates included in the analysis

Variable	VIF
Boma type	1.015077
Season	1.289206
Distance to road	1.593272
Distance to river	1.243011
Distance to protected area	1.463432
Proportion of agriculture	1.591088
NDVI	1.343720

**Table A2:** Random effect intercepts for “village” and “year” for the “Raid attempt” and the “Raid success” models for spotted hyena attacks in livestock bomas

	Intercept for attempt model	Intercepts for success model
Village		
Emboreet	−0.024548813	−0.02769066
Engaruka	−0.059274514	0.13754701
Esilalei	−0.00617071	−0.06152345
Kakoi	0.006565166	−0.02526322
Lolkisale	−0.015124702	−0.01830449
Losirwa	−0.024880434	−0.02254452
Makuyuni	0.178386579	0.08913596
Minjingu	0.021656667	0.10627708
Mswakini	0.005386356	−0.02753653
Naiti	−0.018415988	0.12069113
Olasiti	−0.09417371	−0.10093245
Oltukai	0.010698369	−0.23062695
Selela	0.033692215	0.10866054
*SD*	0.1689	0.2659
Year		
2009	0.003339861	−0.4789217
2010	0.275638011	0.3539537
2011	−0.676206652	−0.3511017
2012	0.30291454	0.3515006
2013	0.184419491	0.2804963
*SD*	0.4316	0.4798

Village	Intercept for raid attempt	Intercept for raid success
Emboreet	−0.0413	−0.0312
Engaruka	−0.0765	0.1648
Esilalei	−0.0050	−0.0625
Makuyuni	0.2718	0.1286
Minjingu	−0.1044	−0.0916
Mswakini	0.0213	−0.0210
Naiti	−0.0249	0.1350
Olasiti	−0.1193	−0.0733
Oltukai	0.0452	−0.2179
Selela	0.0576	0.1244
*SD*	0.1120	0.2794

Year	Intercept for attempt model	Intercepts for success model
2009	0.0411	−0.4069
2010	0.2067	0.2288
2011	−0.7526	−0.2860
2012	0.3250	0.3309
2013	0.2899	0.2663
*SD*	0.4853	0.4330
